# Genetic etiology and pregnancy outcomes of fetal hyperechoic kidneys: a retrospective analysis

**DOI:** 10.3389/fped.2025.1496381

**Published:** 2025-08-20

**Authors:** Meiying Cai, Na Lin, Ziheng Xiao, Hailong Huang, Lin Zheng, Liangpu Xu

**Affiliations:** ^1^ Medical Genetic Diagnosis and Therapy Center, Fujian Maternity and Child Health Hospital College of Clinical Medicine for Obstetrics & Gynecology and Pediatrics, Fujian Medical University, Fujian Key Laboratory for Prenatal Diagnosis and Birth Defect, Fuzhou, China; ^2^ The Clinical Laboratory Center of the Second Affiliated Hospital of Fujian Medical University, Quanzhou, Fujian, China; ^3^ The Graduate School of Fujian Medical University, Fuzhou, Fujian, China

**Keywords:** fetal hyperechoic kidneys, chromosomal microarray analysis, whole-exome sequencing, prenatal ultrasonography, genetic diagnosis in pregnancy

## Abstract

**Background:**

Fetal hyperechoic kidney is an important soft marker in prenatal ultrasonography; however, the causes of this phenomenon are unclear. Therefore, we analyzed genetic diagnosis results, assessed pregnancy outcomes, and conducted postnatal follow-up to provide evidence for prenatal eugenics.

**Methods:**

We retrospectively analyzed data from 94 cases with fetal hyperechoic kidneys identified between November 2017 and January 2024. Chromosome karyotyping and chromosomal microarray analysis (CMA) were performed on fetuses displaying this phenotype on prenatal ultrasound. For cases with normal results from karyotyping and CMA, whole-exome sequencing (WES) was applied.

**Results:**

Among 94 fetuses with hyperechoic kidneys, five were not subject to chromosome karyotyping owing to gestational age constraints, and one sample failed to culture. Of the remaining 88, karyotyping helped detect six cases with abnormal karyotypes. Among 94 fetuses with hyperechoic kidneys, CMA analysis was performed on 90 fetuses, and 17 cases of abnormal copy number variations (CNVs) were detected. Furthermore, among 82 fetuses with normal karyotypes, 10 additional abnormal CNVs were identified. WES, performed on 13 fetuses with normal chromosomal karyotypes and CMA, helped identify three cases of mutations in *HNF1B*, *NPHP3*, and *KMT2D*. Follow-up of 94 fetuses indicated that 16 were lost to follow-up. Of the 78 followed-up, 25 pregnancies were terminated, and one fetus died *in utero*. Post-birth follow-up of 52 live births revealed an adverse outcome incidence of 3.85% (2/52), consisting of one neonatal death within 24 h and one case of intellectual disability.

**Conclusions:**

CMA is recommended when prenatal ultrasound indicates fetal hyperechoic kidneys. For fetuses with normal CNVs and persistent hyperechoic kidneys, WES is advisable to exclude rare monogenic disorders. In cases of hyperechoic kidneys alongside other ultrasound abnormalities, the live birth rate and prognosis tend to be poor; thus, early genetic screening is essential to guide pregnancy management effectively.

## Introduction

Fetal hyperechoic kidney is a common soft marker of prenatal ultrasound abnormalities, defined by a renal echo more brighter than the liver (1). This condition may result from congenital urinary system malformations, chromosomal abnormalities, monogenic mutations, normal kidney variations, or other conditions (2–6). Clinical manifestations and prognoses differ depending on the underlying causes. Currently, hyperechoic kidney is primarily categorized into isolated and non-isolated types, depending on the presence of additional abnormalities (7). Isolated hyperechoic kidney may only manifest as hyperechoic kidney prior to delivery and could be associated with renal cystic lesions, abnormal amniotic fluid volume, and renal volume changes (8). Non-isolated hyperechoic kidney frequently results in more severe conditions that may lead to neurological and cardiovascular malformations, consequently increasing the rate of pregnancy termination (9). Yulia et al. (9) recommended chromosome karyotyping analysis, chromosomal microarray analysis, or genetic testing when fetal hyperechoic kidney is observed in prenatal testing after 20 weeks of pregnancy, which is consistent with current prenatal guidance protocols. Genetic testing and postpartum follow-up of fetuses with hyperechoic kidneys enhance our understanding of the various causes of hyperechoic kidney.

In prenatal examinations, numerous cases with fetal hyperechoic kidney, such as those with abnormal chromosome numbers, are encountered. These cases do not alter the adverse outcomes of fetal illness or death, whether occurring intrauterine or postnatally. Therefore, screening for chromosomal and genetic abnormalities upon detection of fetal hyperechoic kidney during prenatal testing is crucial. Accordingly, we retrospectively analyzed data from ultrasound examinations, genetic factors, and postnatal follow-ups for 94 fetuses with hyperechoic kidneys to provide accurate etiological diagnoses for clinical practice, comprehensive genetic counseling, prognostic analysis, postnatal medical guidance, and to prevent unnecessary terminations of pregnancy.

## Materials and methods

### Patient data

A retrospective analysis was conducted on 94 cases with fetal hyperechoic kidneys identified at Fujian Maternal and Child Health Hospital from November 2017 to January 2024. Cases further treated at a prenatal diagnosis center that provided informed consent for interventional prenatal diagnosis were included. The mean age of the pregnant women was 29.6 years (range, 23–41 years); the mean gestational age was 25.83 weeks (range, 15–34 + 4 weeks). According to whether other ultrasound abnormalities were combined, cases were categorized into isolated (16 cases) and non-isolated (78 cases) hyperechoic kidney groups. Postpartum follow-up included collection of pregnancy information, results of postpartum ultrasound examinations, and determination of the need for surgical treatment. This is a secondary analysis of data (Ethics Approval No.: 2014042), where all participants provided written informed consent explicitly permitting future research use of their de-identified data. The current study was separately approved by the Ethics Committee of Fujian Maternal and Child Health Hospital.

### Ultrasound examination

**Figure 1:**
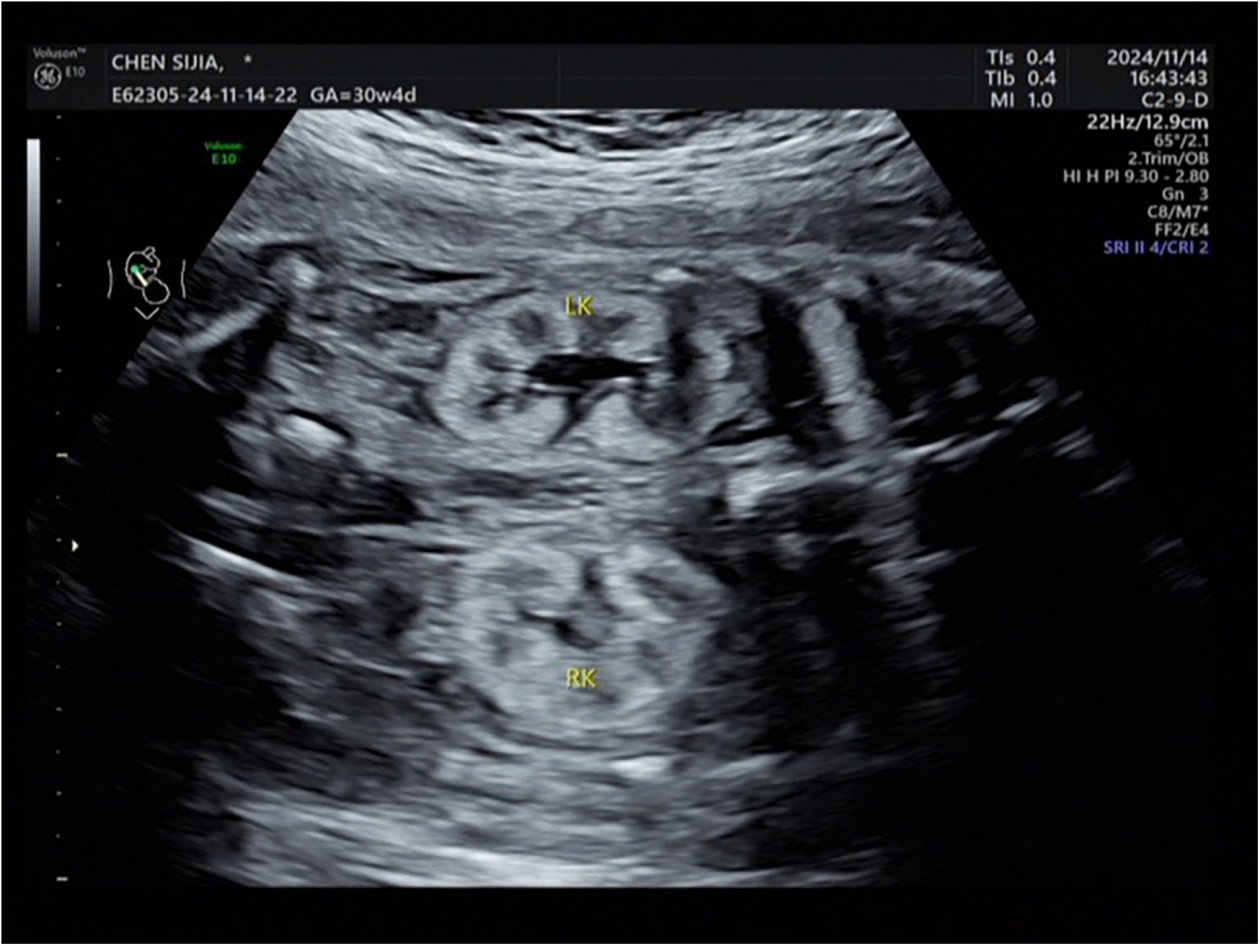
Intrauterine ultrasound phenotype of fetal hyperechoic kidney.

The Voluson E8 ultrasound diagnostic instrument from GE in the United States, with an abdominal probe frequency of 2.0–5.0 MHz. was employed. A systematic examination of the entire body and accessory structures of the fetus was performed along with routine biological measurements. Examination of fetal kidneys included assessments of kidney size and shape, echo, collecting system, a clear boundary between the cortex and medulla, and the presence of cysts. The criterion for diagnosing fetal hyperechoic kidney was renal echo during mid-to-late pregnancy brighter than the liver (Figure 1). The deepest vertical pocket and amniotic fluid index were used to evaluate amniotic fluid volume during mid and late pregnancy, respectively. Cases were categorized as isolated or non-isolated hyperechoic kidney based on the presence of additional abnormalities.

### Preparation and analysis of chromosome karyotypes

Under the guidance of B-ultrasound, we performed amniocentesis to extract amniotic fluid from pregnant women. Amniotic fluid specimens were placed in two sterile centrifuge tubes and centrifuged at 2,000 rpm for 10 min. The supernatant was discarded, and the precipitate was inoculated into two bottles of 4 ml amniotic fluid cell culture medium. The culture was then incubated at 37°C in a 5% CO_2_ incubator for 8 days. After the cells grew well, the medium was changed, and the culture was continued for an additional day. Upon observing multiple cell clones under an inverted microscope and refractive amniotic fluid cells constituting ≥90% of the cloned cells, colchicine was added to arrest the culture. The adherent cells were digested with trypsin, and hypotonic, fixed, droplet, band, and Giemsa staining were performed. We counted ≥20 split cells cultured in double lines per sample, analyzed ≥5 split cells, and increased the count when chimerism was observed. The karyotype results are described according to the requirements detailed in the reference (10).

### CMA technical standard operating procedure

Genomic DNA extraction was performed on fetal samples using the column method with the Qiamp DNA Blood MiniKit (Qiagen, Germany). After extraction, the purity and concentration of the DNA were measured using a NanoDrop 2000 ultra microspectrophotometer. Employing the Affymetrix CYTOSSCAN 750K single nucleotide polymorphism microarray detection platform (Affymetrix), 300 ng of genomic DNA was subjected to enzymatic digestion, ligation, PCR, purification, fragmentation, labeling, hybridization, washing, and scanning according to the standard experimental procedure. Analysis was performed using Chromosome Analysis Suite software version 4.2. Interpretation involved a comprehensive analysis of the Online Mendelian Inheritance in Man (OMIM) database, Clinical Genomic Variation Database (ClinVar), DECIPHER database, Database of Genomic Variants, and Clinical Genomic Resources (ClinGen). Following guidelines from the American Society for Medical Genetics and Genomics (11, 12), CNVs were classified into five levels: benign CNV, pathogenic CNV, likely pathogenic CNV, likely benign CNV, and variant of uncertain significance (VUS).

### WES technical standard operating procedure

WES was performed by Beijing BioChain Medical Laboratory. Fetal DNA was cut into millions of small DNA fragments to construct a genomic library, obtain exon sequences using targeted hybridization probes, and sequence the DNA. Following sequencing, the raw data were aligned using BWA software. Mutations, including single nucleotide polymorphisms (SNPs), insertions, and deletions, were identified and analyzed using GATK and VarScan software for detection and annotation. Annovar software was used to annotate variant sites from external databases and evaluate the impact of target sequence mutations. Mutations detected using whole-exome technology were classified according to the guidelines of the American College of Medical Genetics and Genomics (ACMG) into pathogenic mutations, suspected pathogenic mutations, mutations of unknown significance, suspected benign mutations, and benign mutations (13). The DNA sequence obtained through the WES of the family was compared with that of the reference human genome hg19, and the coverage and sequencing quality of the target area were evaluated. Bioinformatics analysis was performed on the variations, and potential pathogenic homozygous and compound heterozygous single nucleotide variations and small variations were screened under quality control standards of target area coverage >99% and average depth >120×. Based on the site alignment of family sequencing data, the genetic patterns of sample variations were analyzed. The median turnaround time for WES results was 25 days (range: 20–28), with results typically available at a median gestational age of 28 weeks (range: 24–34).

### Pregnancy outcome and postnatal follow-up

The internal clinical information registration system of the hospital and telephone follow-ups were used to track fetal pregnancy outcomes and the postnatal growth and neurobehavioral development of infants. Outcomes included live births, fetal deaths *in utero*, pregnancy terminations, spontaneous abortions, and infant deaths. Follow-ups, including evaluations of postpartum imaging, surgical interventions, effectiveness of the surgery, and growth and intellectual development of the neonates, were conducted on all cases after birth.

### Statistical analysis

The obtained data were analyzed using SPSS 25.0 statistical software, and Fisher's exact probability method was used for rate comparisons. All statistical tests were conducted using a two-sided design, with a significance level of *α* = 0.05. A *P* < 0.05 is considered statistically significant.

## Results

### Chromosome karyotype analysis

Among the 94 fetuses with hyperechoic kidneys, five were excluded from karyotype analysis owing to advanced gestational age, and one sample failed to culture. We analyzed karyotypes in 88 cases and detected six abnormal karyotypes (6.82%, 6/88), including four cases with abnormal chromosome numbers (three cases of trisomy 21 and one case of XXY) and two cases with abnormal chromosome structures. Chromosomal structural abnormalities comprised one case of a large segment deletion [46, X, del (X) (q28)] and one case of an unbalanced chromosomal translocation [46, X, add (13) (p11)]. In follow-up, except for cases carrying 46, X, del (X) (q28) who refused follow-up, all pregnancies involving the five fetuses with identified karyotype abnormalities were terminated.

### CMA analysis

Among the 94 cases, four were not assessed, and the results from 90 fetuses revealed 17 with abnormal CNVs (18.89%, 17/90), including 12 pathogenic CNVs (13.33%, 12/90), two likely pathogenic CNVs (2.22%, 2/90), and three VUS (3.33%, 3/90). Identified abnormalities included three cases of aneuploidy (two trisomy 21, one 47, XXY), six cases of 17q12 microdeletion, two cases of 1p36.33p36.32 microdeletion, and one case each of 16p11.2 microdeletion, Xq28 deletion, 13q31.1q34 duplication, 16q22.2q23.2 microdeletion, 8q11.23q24.3 uniparental disomy, and 2p25.3p11.2 uniparental disomy (Tables 1, 2). In the follow-up of pregnancy outcomes of 17 fetuses with hyperechoic kidneys carrying abnormal CNVs, follow-up was refused for four cases, six pregnancies were terminated, and seven fetuses with normal clinical phenotypes were successfully followed up after birth.

**Table 1:** Clinical characteristics of abnormal CNV in 17 fetuses with hyperechoic kidney.

Case	Ultrasonography	CMA	Classification	Outcome
1	hyperechoic kidney, enhanced bowel echo, strong left ventricular echo	arr[hg19] (21)x3	P	TP
2	hyperechoic kidney, enhanced bowel echo, cardiac anomalies	arr[hg19] (21)x3	P	TP
3	hyperechoic kidney, cardiac anomalies	arr[hg19] XXY	P	TP
4	hyperechoic kidney, strong left ventricular echo	arr[hg19]Xq28 (147550751_155233098)x1	P	Loss follow-up
5	hyperechoic kidney, enhanced bowel echo, single umbilical artery, nasal bone dysplasia	arr[hg19]13q31.1q34 (83,191,742-115,107,733)x3	P	TP
6	hyperechoic kidney, enhanced bowel echo	arr[hg19]17q12 (34,822,465-36,307,773)x1	P	Intellectual disability
7	hyperechoic kidney, deepening of alpha wave notches in venous catheter blood flow spectrum	arr[hg19]17q12 (34,822,465-36,418,529)x1	P	TP
8	hyperechoic kidney	arr[hg19]17q12 (34,823,294-36,410,720)x1	P	Normal
9	hyperechoic kidney, bilateral choroid plexus cyst, strong left ventricular echo, mild tricuspid regurgitation	arr[hg19]17q12 (34,822,465-36,307,773)x1	P	Normal
10	hyperechoic kidney	arr[hg19]17q12 (34,822,465-36,243,365)x1	P	Loss follow-up
11	hyperechoic kidney, strephenopodia	arr[hg19]17q12 (34,822,466-36,404,104)x1	P	Loss follow-up
12	hyperechoic kidney, fetal cerebral ventriculomegaly	arr[hg19]1p36.33p36.32 (849,466-4,894,800)x1,11p15.5p15.4 (230,680-8,918,951)x3	P	TP
13	hyperechoic kidney, etal cerebral ventriculomegaly	arr[hg19]1p36.33p36.32 (849,467-4,894,800)x1	LP	Loss follow-up
14	hyperechoic kidney, single umbilical artery	arr[hg19]16p11.2 (29,428,531-30,177,916)x1	LP	Normal
15	hyperechoic kidney	arr[hg19]8q11.23q24.3 (55,365,228-146,292,734)x2 hmz	VUS	Normal
16	hyperechoic kidney, cardiac anomalies, fetal growth restriction	arr[hg19]2p25.3p11.2 (50,813-87,053,152) hmz arr[hg19]2q11.1q37.3 (95,550,957-242,773,583) hmz	VUS	Normal
17	hyperechoic kidney, enhanced bowel echo, cardiac anomalies	arr[hg19]16q22.2q23.2 (71,463,698-9,614,082)x3	VUS	Normal

P, pathogenic; LP, likely pathogenic; TP, termination of pregnancy; VUS, variant of uncertain significance.

**Table 2:** Detailed classification of pathogenic variants in CMA.

Variant type	Genomic coordinates (hg19) size/position	Size/position	Encompassed genes/key regions	Known phenotypic associations	ACMG classification evidence
Xq28 deletion	arr[hg19]Xq28 (147550751_155233098)x1	7.68 Mb	MECP2, FLNA, L1CAM	X-linked intellectual disability, Rett syndrome, periventricular heterotopia	PVS1, PM2, PP5
13q31.1q34 triplication	arr[hg19]13q31.1q34 (83,191,742-115,107,733)x3	31.92 Mb	SOX21, EFNB2	Growth overgrowth, macrocephaly, developmental delay (13q triplication syndrome)	PS3, PM1
17q12 deletion	arr[hg19]17q12 (34,822,465-36,307,773)x1	1.42–1.60 Mb	HNF1B (key gene)	Renal cysts and diabetes syndrome (RCAD)	PVS1, PS4
1p36.33p36.32 deletion	arr[hg19]1p36.33p36.32 (849,466-4,894,800)x1	4.05 Mb	MMP23B, SKI	1p36 deletion syndrome (intellectual disability, epilepsy, characteristic facies)	PS1, PM2
11p15.5p15.4 triplication	arr[hg19]11p15.5p15.4 (230,680-8,918,951)x3	8.69 Mb	IGF2, H19 (imprinted region)	Beckwith-Wiedemann syndrome (BWS)	PS3 (LOI confirmed), PM1
16p11.2 deletion	arr[hg19]16p11.2 (29,428,531-30,177,916)x1	749 kb	SH2B1	Autism spectrum disorder, obesity	PS4

Conventional karyotype analysis and CMA both helped effectively detect chromosomal numerical abnormalities and large fragment deletions or duplications. We employed these methods to identify two simultaneous cases of trisomy 21: one involved an abnormal sex chromosome number, another a chromosome duplication, and a third involved a chromosome deletion. Routine karyotype analysis revealed trisomy 21 in a patient who did not undergo CMA testing. CMA helped detect an additional 10 abnormal CNVs in 82 fetuses with normal chromosome karyotypes, including seven pathogenic CNVs, one likely pathogenic CNV, and two VUS. This increased the detection rate of chromosomal abnormalities by 12.20% (10/82). Additionally, CMA helped detect two additional abnormal CNVs in four fetuses without chromosomal karyotyping, including one harboring pathogenic CNV and one with a likely pathogenic CNV.

### WES results

Among the 94 fetuses with hyperechoic kidneys, 13 cases with normal conventional karyotype and CMA results were further subjected to WES; two cases of pathogenic mutations and one likely pathogenic gene mutation involving genes, *HNF1B*, *NPHP3*, and *KMT2D*, were identified (Tables 3, 4). The key clinical distinction between fetal hyperechoic kidneys with normal vs. abnormal whole-exome sequencing (WES) results primarily manifests in the presence of concurrent extrarenal anomalies (Table 5).

**Table 3:** Clinical characteristics of abnormal genes in three fetuses with enhanced renal echo.

Case	Ultrasonography	Gene	Variation	Inheritance	Origin	Classification	Outcome
1	hyperechoic kidney	*HNF1B*	Chr17:36064918-36064921 c.1339+3_1339+6 delAAGT	AD	*denovo*	LP	Normal
2	hyperechoic kidney, ventriculomegaly, thickened nuchal translucency	*NPHP3*	Chr3:132403565 rs746849675 c.3402_3403del p.A1135Sfs*5 Chr3:132438657 c.411delT p.Q138Rfs*11	AR	Paternal, maternal	P	TP
3	hyperechoic kidney, ventriculomegaly	*KMT2D*	Chr12:49434993 c.6547_6560del p.Y2183Pfs*14	AD	*denovo*	P	TP

AD, autosomal dominant inheritance; AR, autosomal recessive inheritance; P, pathogenic; LP, likely pathogenic; TP, termination of pregnanc.

**Table 4:** Detailed classification of pathogenic variants in WES.

Gene	Genomic coordinates (hg19) size/position	Variant type	Encompassed genes/key regions	Known phenotypic associations	ACMG classification evidence
HNF1B	Chr17:36064918-36064921 c.1339+3_1339+6 delAAGT	Splice site deletion	BRCA1 (intron 9)	Breast/ovarian cancer (disrupts canonical splice site)	PVS1, PM5
NPHP3	Chr3:132403565 rs746849675 c.3402_3403del p.A1135Sfs*5	Frameshift variant	VHL (exon 3)	Von Hippel-Lindau syndrome (renal cell carcinoma risk)	PVS1, PM2, PP3
NPHP3	Chr3:132438657 c.411delT p.Q138Rfs*11	Frameshift variant	COL7A1 (exon 3)	Dystrophic epidermolysis bullosa	PVS1, PM2, PP1 (segregation confirmed)
KMT2D	Chr12:49434993 c.6547_6560del p.Y2183Pfs*14	Frameshift variant	BRCA2 (exon 11)	Breast/ovarian cancer	PVS1, PS4 (PMID:456789)

**Table 5:** Clinical characteristics of other renal findings and other anomalies for fetal hyperechoic kidneys with normal vs. abnormal WES.

Characteristic	WES normal (*n*)	WES abnormal (*n*)
Isolated fetal hyperechoic kidney	20% (2/10)	0 (0/3)
Other renal findings	0 (0/10)	0 (0/3)
Oligohydramnios	0 (0/10)	0 (0/3)
Extrarenal anomalies	80% (8/10)	100% (3/3)

### Analysis of genetic abnormalities in each group with hyperechoic kidney

We categorized 94 cases into isolated and non-isolated fetal hyperechoic kidneys groups based on the presence of other ultrasound abnormalities. In the isolated fetal hyperechoic kidneys group (including 16 cases), we detected two cases of pathogenic CNV, one VUS, and one potential pathogenic gene mutation, resulting in a genetic abnormality detection rate of 25.00% (4/16). In the non-isolated fetal hyperechoic kidneys group (including 78 cases), we found 10 cases of pathogenic CNVs, two cases of potentially pathogenic CNVs, two cases of VUS, and two cases of pathogenic gene mutations. The detection rate of genetic abnormalities was 20.51% (16/78), with no statistically significant difference in the detection rate of genetic abnormalities between the two groups (*P* > 0.05).

### Pregnancy outcome

Out of 94 cases of fetuses with hyperechoic kidneys, 78 completed pregnancy outcome follow-up; 16 were lost to follow-up. Of the followed-up cases, pregnancies were terminated in 25 cases, one fetal death occurred *in utero*, and 52 live births were recorded.

### Postnatal follow-up

After follow-up observation of 52 live births, 2 cases experienced adverse outcomes post-birth, with an incidence rate of 3.85% (2/52). One fetus died within 24 h of birth despite normal genomic testing and an intrauterine ultrasound phenotype of hyperechoic kidney and oligohydramnios. Another exhibited fetal intellectual developmental delay post-birth with CMA, revealing a 17q12 microdeletion and intrauterine ultrasound phenotype of hyperechoic kidney and strong intestinal echo. Among the remaining 50 live births, 21 refused post-birth ultrasound follow-up, 24 had normal renal ultrasound results, and six maintained the same ultrasound conditions as before (these six cases within the non-isolated hyperechoic kidney).

## Discussion

Fetal hyperechoic kidneys is an important manifestation of congenital renal dysplasia, with a detection rate of approximately 0.16% (1, 14). While often a nonspecific normal variation, hyperechoic kidney also serves as a clinically instructive ultrasound indicator (15). We categorized hyperechoic kidney into isolated and non-isolated types based on the presence of other ultrasonic abnormalities. Hyperechoic kidney is frequently detected in trisomy 21, trisomy 18, and trisomy 13 syndromes (16). The detection rate of chromosomal abnormalities in isolated fetal hyperechoic kidneys ranges from 21.4%–28.1%, with trisomy 9, trisomy 13, and 17q12 chromosome microdeletion as predominant pathogenic factors (16). In our study, we found that the detection rate of chromosomal abnormalities in isolated hyperechoic kidney was 25.00% (4/16), aligning with that reported in the literature.

Our findings highlight 17q12 microdeletion as a primary genetic pathogenic factor for fetal hyperechoic kidneys (6.52%, 6/92), often resulting from low copy number non-allelic homologous recombination (17). The core pathogenic gene within the 17q12 microdeletion is the hepatocyte nuclear factor-1β (*HNF1B*), a DNA-binding transcription factor essential for normal renal development. Deletion of *HNF1B* primarily contributes to simple renal echo enhancement (11–13, 18, 19). In this study, we observed genetic abnormality detection rates of 25% (4/16) in isolated hyperechoic kidney cases and 20.51% (16/78) in non-isolated cases, with no significant difference between them (*P* > 0.05). Consequently, we advocate for karyotype analysis and CMA irrespective of additional ultrasonic abnormalities when fetal hyperechoic kidney is evident.

Recent advances in WES have enhanced the diagnosis of monogenic diseases, identifying specific gene mutation sites causing fetal hyperechoic kidney (16, 20). Shuster et al. (7) identified an *HNF1B* mutation linked to hyperechoic kidney. Additional gene mutations, such as those found in *PKHD1*, *PKD*, *PAX2*, and RET, have been implicated in recent findings related to hyperechoic kidney (21). We conducted WES on 13 of 94 cases, discovering mutations in *HNF1B*, *NPHP3*, and *KMT2D*. *HNF1B* is predominantly associated with renal tubulointerstitial nephropathy, which is characterized by renal interstitial fibrosis, tubular atrophy, and basal membrane thickness alteration (4). *NPHP3* is implicated in nephropathy involving significant yet nonspecific pathologies, with occasional extra-renal system involvement (22). Variations in *KMT2D* are associated with Kabuki syndrome (KS) (23). KS is a group of autosomal dominant genetic disorders characterized by distinctive facial and skeletal abnormalities, abnormal skin texture, congenital visceral abnormalities, postnatal growth restriction, and mild-to-moderate intellectual impairment (24). In this study, we observed that the ultrasound phenotypes of a fetus with *HNF1B* mutation included hyperechoic kidney and polycystic kidney, whereas the ultrasound phenotypes of the fetus with *NPHP3* and *KMT2D* gene mutations included hyperechoic kidney and abnormal nervous system structure. Collectively, we recommend comprehensive WES to exclude rare monogenic genetic diseases in fetuses displaying hyperechoic kidney with normal CMA and chromosomal karyotype analysis.

The outcome of isolated hyperechoic kidney is significantly better than that of non-isolated hyperechoic kidney, with most cases of isolated enhancement showing favorable outcomes (9, 25). While our results showed no significant difference in genetic detection rates between isolated vs. non-isolated renal hyperechogenicity groups, clinically we observed better overall outcomes in cases with isolated findings. This suggests that even when genetic conditions are present, those with isolated renal manifestations may have less severe phenotypic expression. The analysis of clinical characteristics in fetal hyperechoic kidneys with normal or abnormal WES showed extrarenal anomalies. In this study, two cases of non-isolated hyperechoic kidney exhibited poor prognosis, which aligns with the findings reported in the literature. We observed that in clinical scenarios where hyperechoic kidney combined with other ultrasound abnormalities, the live birth rate and prognosis are generally poor. Early screening for genetic abnormalities is imperative to guide pregnancy management effectively, inform risk assessment, and facilitate informed decision-making regarding the continuation of pregnancy. Moreover, not all cases of hyperechoic kidney indicate underlying disease; therefore, meticulous observation and follow-up are crucial to accurately assess prognosis and prevent unnecessary terminations of pregnancy.

Consistent with previous prenatal cohorts, our study identified a VUS detection rate of 3.33% through CMA (26). In prenatal genetic diagnosis, the interpretation of VUS remains a critical challenge, primarily due to: limited parental verification, heterogeneity in detection platforms, and divergent classification criteria across laboratories. This study identified 3 VUS cases (representing 3.33% of all detected variants), all of which lacked parental origin data due to refusal of follow-up testing. Future studies should prioritize: longitudinal tracking of such VUS through international databases, functional assays to assess the impact of non-coding VUS, standardized reporting frameworks for prenatal VUS.

However, our study has the following limitations: retrospective nature over an extended period, possible omission of some cases, and selective application of WES owing to its high costs. While cost remains a primary barrier to WES implementation, the temporal constraints may represent an equally significant limitation in clinical practice. Additionally, the follow-up period for live births was insufficiently brief; therefore, we plan extended follow-up to acquire more accurate clinical information in the future.

## Conclusions

When the fetal intrauterine ultrasound phenotype presents as a hyperechoic kidney, chromosome karyotype analysis, and CMA examination should be performed, regardless of the presence of other ultrasound abnormalities. In cases with normal karyotyping and CMA results but persistent hyperechoic kidney, we recommend further testing to exclude rare monogenic inherited diseases. The live birth rate and prognosis of fetuses exhibiting hyperechoic kidney alongside abnormal ultrasound structures are often unfavorable. Therefore, we recommend prioritizing early screening for genetic abnormalities to effectively guide pregnancy management, accurately inform risk, and facilitate informed decisions on continuing the pregnancy. Furthermore, not all hyperechoic kidney phenotypes indicate disease; hence, close observation and follow-ups should be conducted to accurately assess prognosis and prevent unnecessary pregnancy terminations.

## Data Availability

The original contributions presented in the study are included in the article/Supplementary Material, further inquiries can be directed to the corresponding authors.
